# Necrotizing Soft Tissue Infection at a Self-Administered Subcutaneous Etanercept Injection Site in an Immunosuppressed Patient With Rheumatoid Arthritis: A Case Report

**DOI:** 10.7759/cureus.100765

**Published:** 2026-01-04

**Authors:** Seigo Tai, Masaaki Takemoto, Yoshihiro Yamamoto, Takaaki Nakano, Toshitaka Ito

**Affiliations:** 1 Department of Emergency Medicine, Shin-Yurigaoka General Hospital, Kawasaki, JPN

**Keywords:** etanercept, necrotizing soft tissue infection, streptococcal toxic shock syndrome, streptococcus pyogenes, subcutaneous injection

## Abstract

Necrotizing soft tissue infection (NSTI) is rapidly progressive and can be fatal; outcomes depend on early recognition and prompt surgical debridement. In immunocompromised patients, local findings and inflammatory responses may be atypical, increasing the risk of delayed diagnosis. Although injections breach the skin barrier, NSTI originating at injection sites can be overlooked as a benign injection-site reaction; procedure-associated NSTI has been reported to have poor outcomes. A woman in her 60s with rheumatoid arthritis was receiving methotrexate, tacrolimus, and prednisolone and had started self-administered subcutaneous etanercept injections four months earlier. One week prior to presentation, she developed discomfort and mild pain in the medial right thigh at her usual injection site, and, presuming it was a routine injection-site reaction, delayed medical attention. She presented with fever and difficulty walking and was transferred to our hospital in septic shock. Examination revealed ill-defined erythema and swelling without overt skin necrosis, but pain was disproportionately severe. Contrast-enhanced computed tomography (CT) showed fascial thickening and deep soft-tissue edema without gas. Given a strong clinical suspicion for NSTI, emergent surgical exploration (finger test) and debridement were performed, confirming necrosis of the subcutaneous tissue and fascia. Group A β-hemolytic streptococcus (*Streptococcus pyogenes*) was isolated from wound and blood cultures, and the clinical course was consistent with streptococcal toxic shock syndrome (STSS). Molecular typing (e.g., emm typing) and laboratory assays proving toxin production were not performed; STSS was diagnosed clinically. The patient required additional debridement, intensive care, and targeted antimicrobial therapy and survived without amputation. She was discharged ambulatory on hospital day 105. This case highlights that, in immunosuppressed patients who self-inject biologic agents, injection-site symptoms should not be dismissed as routine reactions when pain is disproportionate or systemic symptoms develop. Early imaging and prompt surgical evaluation are essential when NSTI is suspected, even if skin findings are subtle.

## Introduction

Necrotizing soft tissue infection (NSTI) is a life-threatening infection involving the skin, subcutaneous tissue, fascia, and sometimes muscle. Mortality remains substantial, and prognosis depends strongly on early diagnosis and prompt surgical debridement. Delayed recognition and delayed source control are consistently associated with worse outcomes [1-4].

The Laboratory Risk Indicator for Necrotizing Fasciitis (LRINEC) score was developed to support risk stratification [5]; however, a subsequent systematic review and meta-analysis demonstrate limited sensitivity [6], and LRINEC should not be used to exclude NSTI. Because time to definitive surgery is a critical determinant of outcomes, early diagnosis and prompt debridement remain fundamental [7,8].

Portals of entry for NSTI are diverse, including minor trauma and surgical wounds; procedure-related entry points such as injections and other invasive procedures are also recognized [1-4,9]. Necrotizing soft tissue infections following injection therapy have been reported to have higher mortality and poorer outcomes compared with other entry mechanisms [9].

Biologic agents (e.g., anti-tumor necrosis factor (anti-TNF) agents) are effective for rheumatoid arthritis. Several case reports have described necrotizing fasciitis in patients receiving etanercept, highlighting that anti-TNF therapy can predispose to severe soft-tissue infections and that early recognition is critical [10-12]. Anti-TNF therapy is also associated with an increased risk of serious infections in rheumatoid arthritis [13-16]. *Streptococcus pyogenes* (group A Streptococcus) can cause fulminant NSTI, and clinical deterioration may be complicated by streptococcal toxic shock syndrome (STSS) [17,18].

We report a case of fulminant streptococcal NSTI developing at the site of self-administered subcutaneous etanercept injection in an immunosuppressed patient with rheumatoid arthritis, initially misattributed to an injection-site reaction and resulting in delayed presentation.

## Case presentation

Patient information

A woman in her 60s with rheumatoid arthritis presented with fever and difficulty ambulating. Her past medical history included chronic kidney disease (stage 3), hypertension, dyslipidemia, and a cerebral aneurysm treated by endovascular embolization.

Medications and treatment history

She was receiving methotrexate 12 mg weekly, tacrolimus 1 mg daily, prednisolone 5 mg daily, and folic acid 5 mg daily. Self-administered subcutaneous etanercept 50 mg weekly was initiated approximately four months prior to presentation.

Social history

She reported long-term tobacco use (approximately 25-60 cigarettes/day for about 40 years) and regular alcohol consumption (approximately half a bottle of champagne nearly daily).

History of present illness

She typically injected etanercept into the medial thigh region. Approximately one week prior to admission, she noticed discomfort and mild pain in the medial aspect of the right thigh at her usual injection site. Assuming this was a routine injection-site reaction, she used over-the-counter medication and did not seek care. The pain progressively worsened, and she developed difficulty walking. One day prior to admission, she experienced decreased appetite, generalized fatigue, diarrhea, and mild abdominal pain. On the day of admission, persistent fever and worsening systemic symptoms prompted presentation to emergency services. At the referring facility, examination revealed erythema, swelling, and severe tenderness of the medial right thigh, and septic shock due to severe soft tissue infection was suspected; therefore, she was transferred to our hospital.

Initial assessment and vital signs

On arrival, she was alert (Japan Coma Scale I-1; Glasgow Coma Scale (GCS) E4V5M6). At the referring facility, she had hypotension and tachycardia (blood pressure (BP) 88/49 mmHg, heart rate (HR) 101 beats/minute). At our hospital arrival, her vital signs were respiratory rate 29 breaths/minute, saturation of peripheral oxygen (SpO₂) 97%, BP 110/58 mmHg (norepinephrine 0.2 µg/kg/minute), HR 108 beats/minute, temperature 37.3°C, and GCS E4V5M6.

Physical examination

**Figure 1:**
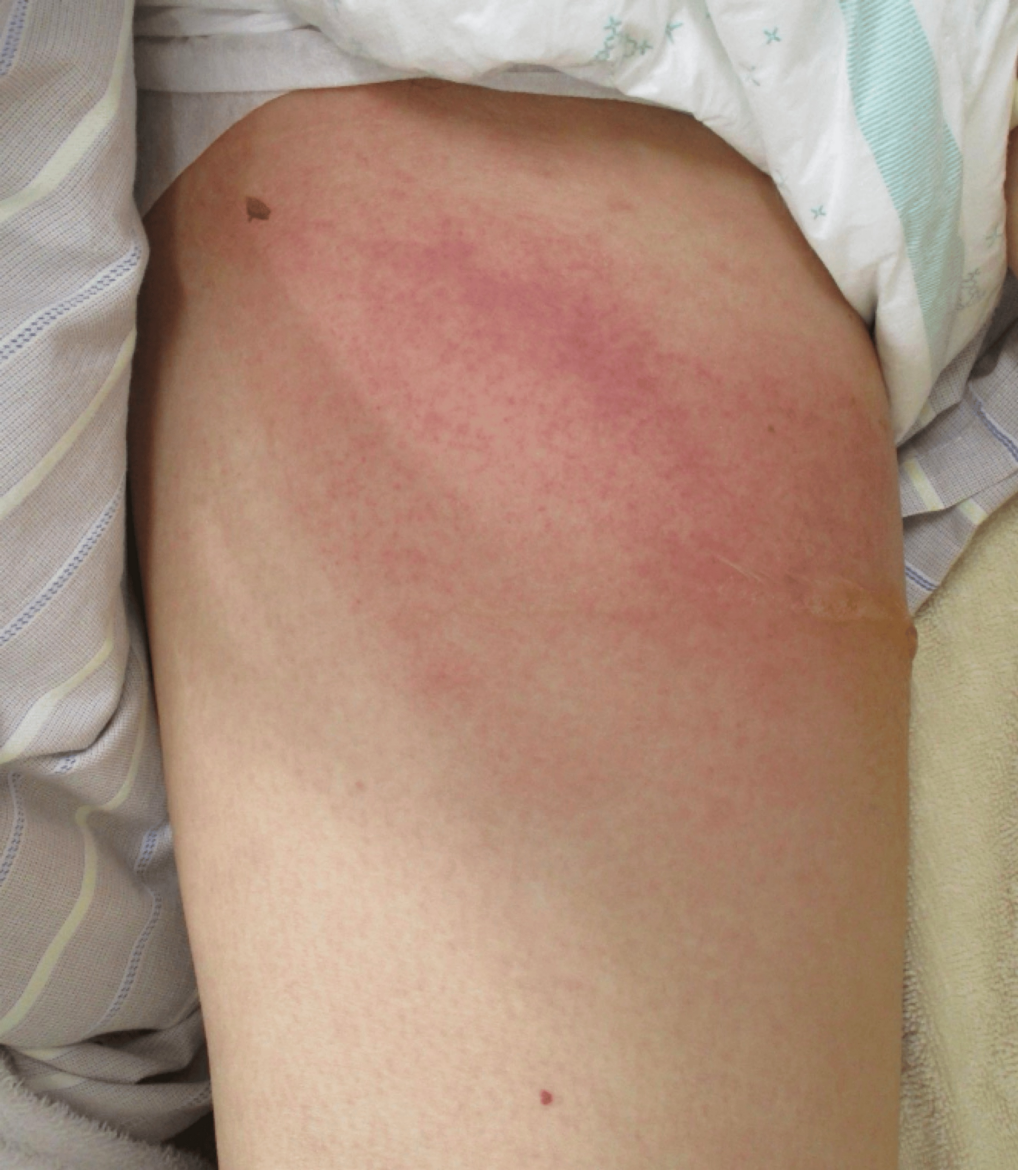
Cutaneous findings on presentation. Ill-defined erythema and swelling on the medial aspect of the right thigh with focal vesiculation and purpuric changes, without overt skin necrosis. The patient reported severe pain disproportionate to the visible findings.

The medial right thigh showed ill-defined erythema and swelling with focal vesiculation and purpuric changes without overt skin necrosis. Lesion dimensions were recorded as erythema/induration 10 cm × 15 cm, with a maximum bulla diameter of 3 cm. Pain was severe and clearly disproportionate to the skin findings (Figure 1).

Laboratory findings

Initial laboratory values are summarized in Table 1. Coagulation studies suggested sepsis-associated coagulopathy. The LRINEC score was 8. Renal dysfunction was evident at presentation (blood urea nitrogen (BUN) 43.10 mg/dL, creatinine 3.230 mg/dL, estimated glomerular filtration rate (eGFR) 12 mL/minute/1.73 m²), consistent with acute kidney injury on chronic kidney disease.

**Table 1:** Initial laboratory data on presentation at the emergency department WBC, white blood cell count; RBC, red blood cell count; Hb, hemoglobin; Ht, hematocrit; Plt, platelet count; Alb, albumin; CK, creatine kinase; T-bil, total bilirubin; AST, aspartate aminotransferase; ALT, alanine aminotransferase; LDH, lactate dehydrogenase; ALP, alkaline phosphatase; BUN, blood urea nitrogen; eGFR, estimated glomerular filtration rate; CRP, C-reactive protein; PaCO₂, arterial partial pressure of carbon dioxide; PaO₂, arterial partial pressure of oxygen; HCO₃⁻, bicarbonate; BE, base excess; PT, prothrombin time; INR, international normalized ratio; APTT, activated partial thromboplastin time; FDP, fibrin/fibrinogen degradation products. Notes: Reference ranges reflect our institutional standards

Parameter	Patient Value	Unit	Reference Range
WBC	5,400	/μL	3,300–8,600
Hemoglobin	10.5	g/dL	11.6–14.8
Platelets	8.8×10^4	/μL	15.8–34.8×10^4
Albumin	1.600	g/dL	3.8–5.2
Creatine kinase	374	U/L	40–150
Total bilirubin	0.510	mg/dL	0.2–1.2
AST	29	U/L	10–40
ALT	18	U/L	7–40
LDH	264	U/L	120–230
ALP	54	U/L	106–322
Amylase	21	U/L	44–132
Uric acid	7.500	mg/dL	2.6–5.5
BUN	43.10	mg/dL	8–20
Sodium	142	mEq/L	138–145
Potassium	4.700	mEq/L	3.6–4.8
Chloride	104	mEq/L	101–108
Glucose	75	mg/dL	70–109
Creatinine	3.230	mg/dL	0.46–0.79
eGFR	12	mL/min/1.73m²	≥60
CRP	27.76	mg/dL	0–0.3
pH	7.453		7.35–7.45
PaCO2	19.50	mmHg	35–45
PaO2	59.20	mmHg	80–100
HCO3-	13.50	mEq/L	22–26
Base excess	-8.700	mEq/L	-2–2
Lactate	2.500	mmol/L	0.5–2
Procalcitonin	>100	ng/mL	<0.05
PT	13.70	sec	10–13
PT activity	79	%	70–130
PT-INR	1.180		0.85–1.15
APTT	32.70	sec	25–35
FDP	14.80	µg/mL	0–5
D-dimer	11.90	µg/mL	0–1
Fibrinogen	556	mg/dL	200–400

Imaging

**Figure 2:**
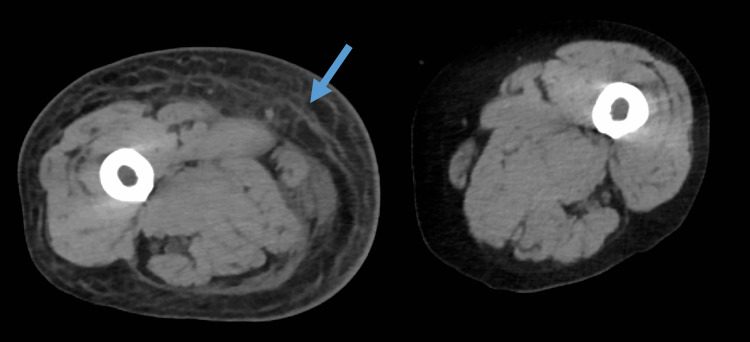
CT on arrival at hospital CT shows fascial thickening and deep soft-tissue edema with fluid tracking along fascial planes in the right medial thigh, without subcutaneous emphysema or gas formation.

Contrast-enhanced CT demonstrated fascial thickening, increased attenuation of subcutaneous fat, fluid tracking along muscle bundles, and obscuration of intermuscular fat planes, consistent with deep soft-tissue inflammation centered at the fascial level (Figure 2). No gas was detected. Absence of gas did not exclude NSTI. Despite renal dysfunction, contrast-enhanced CT was performed after risk-benefit assessment because rapid delineation of deep soft-tissue involvement was considered critical for time-sensitive decision-making in septic shock.

Treatment and clinical course

**Figure 3:**
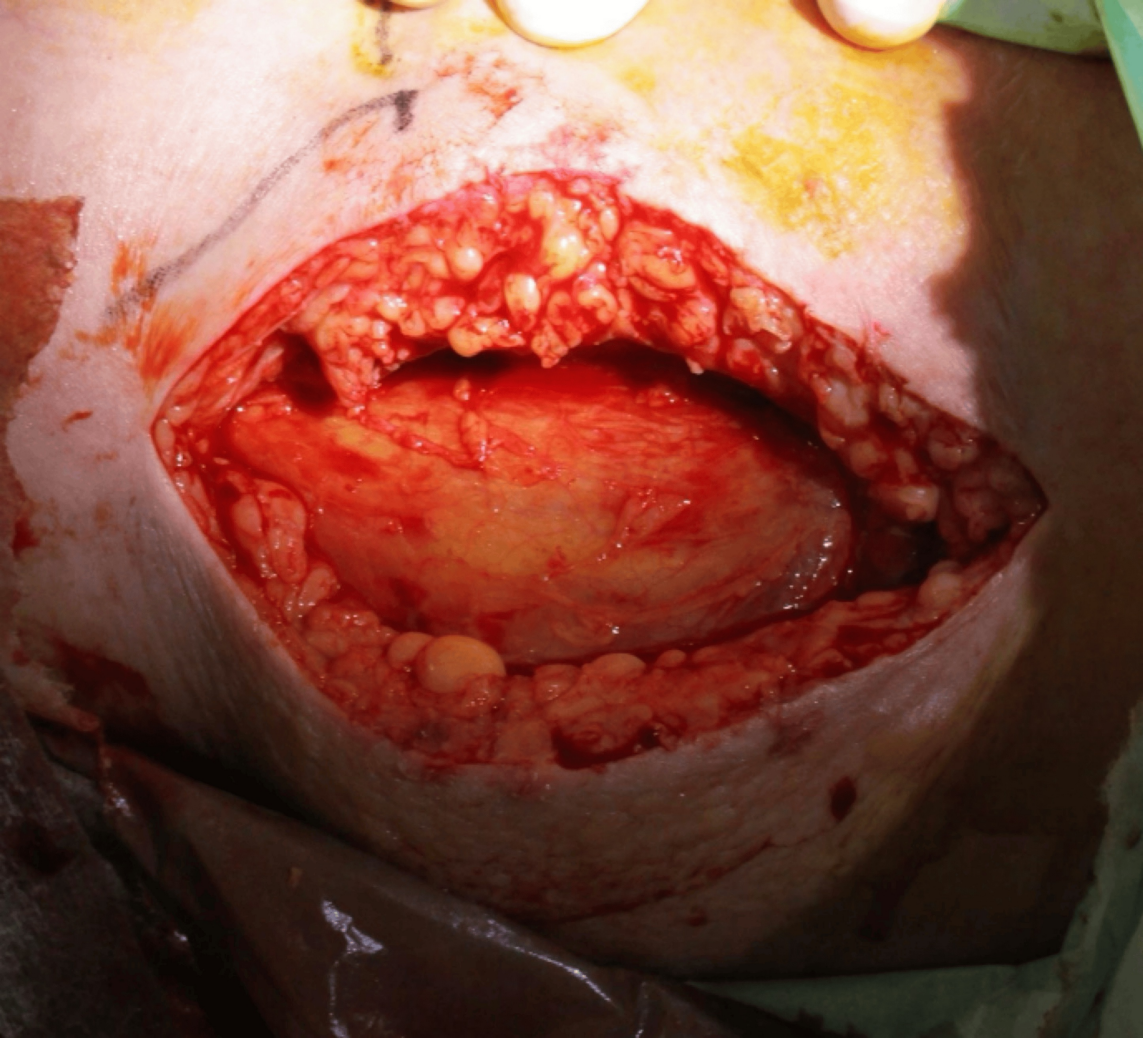
Intraoperative findings (finger test). Exploration reveals gray, dishwater-like fluid. The fascia is friable and easily separated by blunt dissection with minimal bleeding, consistent with NSTI. Necrotic subcutaneous tissue and fascia were debrided, and the wound was left open.

Given septic shock and high clinical suspicion for NSTI, aggressive fluid resuscitation and continuous norepinephrine infusion were initiated. Empiric broad-spectrum antibiotics (meropenem, vancomycin, and clindamycin) were started in accordance with severe SSTI/NSTI management principles [2]. Emergency surgical exploration was performed for diagnostic confirmation and source control. A finger test under local anesthesia revealed gray, dishwater-like fluid. The fascia was friable and easily separated by blunt dissection with minimal bleeding, consistent with NSTI (Figure 3).

Extensive debridement of necrotic subcutaneous tissue and fascia was performed, the wound was irrigated, and left open. The patient was managed postoperatively in the intensive care unit.

Histopathology was not performed because emergent source control was prioritized. Group A β-hemolytic streptococcus (*S. pyogenes*) was isolated from wound and blood cultures. With hypotension and multiorgan dysfunction, the clinical picture was consistent with STSS. Molecular typing (e.g., emm typing) and laboratory assays proving toxin production were not performed; toxin production was not proven, and STSS was diagnosed clinically.

After susceptibility results became available, antimicrobial therapy was de-escalated to penicillin G plus clindamycin in line with guidance for invasive group A streptococcal infection/toxin-mediated disease. Residual necrosis was suspected clinically, and additional debridement was performed on hospital day 2. Thereafter, necrosis did not progress, and granulation tissue developed. Shock, coagulopathy, and acute kidney injury improved, and limb amputation was avoided. After prolonged wound management and rehabilitation, the patient was discharged ambulatory without assistance on hospital day 105.

## Discussion

This case underscores three clinically important points: (i) NSTI may arise at injection sites, (ii) immunosuppression and injection-site reactions can delay recognition, and (iii) early surgical evaluation remains essential even when skin findings appear mild.

Portals of entry and injection-associated NSTI

NSTI typically follows disruption of the skin barrier, enabling pathogens to invade deep tissues and spread rapidly along fascial planes [1,3,4]. While minor trauma and surgical wounds are common portals of entry, procedure-related entry points such as injections, punctures, and other invasive procedures are recognized [1-4,9]. Injections breach the skin barrier and can theoretically introduce pathogens into subcutaneous tissue or near the fascia [3,9]. Procedure-associated NSTI has been reported to have poorer outcomes compared with other entry mechanisms [9]. In the present case, early symptoms localized to the patient’s usual etanercept injection site, supporting injection-site entry as the most plausible portal.

Comparison with prior etanercept-associated necrotizing fasciitis reports

Several case reports have described necrotizing fasciitis in patients receiving etanercept, including a dermatomyositis patient in whom MRI facilitated early diagnosis and timely debridement with a favorable outcome [10], a pediatric patient receiving etanercept and cyclosporine for macrophage activation syndrome [11], and a rheumatoid arthritis patient with shoulder involvement while on etanercept [12]. These reports collectively emphasize that anti-TNF therapy can predispose to severe soft-tissue infections and that early recognition is critical. Our case differs in that the suspected portal of entry was the self-injection site itself, the pathogen was *S. pyogenes* with a clinical course consistent with STSS, and the patient delayed seeking care for approximately one week after localized symptoms, culminating in septic shock requiring urgent surgical source control.

Injection-site selection, rotation, and patient education

The Japanese package insert for etanercept states that injection sites (thigh, abdomen, or upper arm) should be rotated and that patients should not self-administer until they receive proper training; importantly, it does not specify medial versus lateral thigh [19]. In this case, the exact reason for medial-thigh injections could not be verified retrospectively; however, family members consistently reported habitual injections into the same medial-thigh area. This uncertainty was acknowledged as a limitation. Nevertheless, this case suggests that clinicians should reinforce injection-site rotation and advise patients, especially those immunosuppressed, to seek prompt evaluation if injection-site pain is progressive or disproportionate, or if systemic symptoms develop.

Diagnostic strategy and the importance of early surgery

NSTI is primarily a clinical diagnosis [1-4]. Imaging can support diagnosis, but the absence of gas does not exclude NSTI [1-4,6]. LRINEC may support risk stratification but should not be used to rule out NSTI [5,6]. Because the patient had septic shock and pain disproportionate to examination findings, prompt surgical exploration was prioritized, and the finger test rapidly confirmed NSTI, enabling immediate debridement [1-4,7,8].

Streptococcal toxic shock syndrome and adjunctive therapy

In this case, *S. pyogenes* was isolated from wound and blood cultures, and the clinical picture was consistent with STSS [17,18]. Molecular typing (e.g., emm typing) and laboratory assays proving streptococcal toxin production were not performed; therefore, toxin production was not proven, and STSS was diagnosed clinically. Adjunctive intravenous immunoglobulin (IVIg) has been investigated for streptococcal toxin-mediated disease and NSTI; however, the evidence remains mixed [20,21].

Limitations

First, molecular typing and laboratory assays proving toxin production were not performed. Second, the exact rationale for medial-thigh injection and the content of injection-site education could not be fully verified retrospectively, although family members consistently reported habitual injections into the same area. Third, histopathology was not performed due to clinical urgency.Finally, because the patient was immunosuppressed with comorbidities, spontaneous skin infection unrelated to the injection cannot be completely excluded; however, the temporal and anatomic association makes the injection site the most plausible portal of entry.

## Conclusions

NSTI can occur at injection sites and may be misinterpreted as a benign injection-site reaction, particularly in immunosuppressed patients. When pain is disproportionate to skin findings or systemic symptoms develop, clinicians should promptly evaluate for NSTI and prioritize early surgical consultation and exploration, even if the cutaneous appearance is subtle.
